# Hypertension Among US Adults by Disability Status and Type, National Health and Nutrition Examination Survey, 2001–2010

**DOI:** 10.5888/pcd11.140162

**Published:** 2014-08-14

**Authors:** Alissa Stevens, Elizabeth Courtney-Long, Cathleen Gillespie, Brian S. Armour

**Affiliations:** Author Affiliations: Elizabeth Courtney-Long, Brian S. Armour, Division of Human Development and Disability, Centers for Disease Control and Prevention, Atlanta, Georgia; Cathleen Gillespie, Division for Heart Disease and Stroke Prevention, Centers for Disease Control and Prevention, Atlanta, Georgia.

## Abstract

The prevalence of hypertension among people with disabilities is not well understood. We combined data from the 2001–2010 National Health and Nutrition Examination Survey to obtain estimates of hypertension prevalence by disability status and type (cognitive, hearing, vision, or mobility limitation) and assess the association between disability and hypertension. Overall, 34% of adults with disabilities had hypertension compared with 27% of adults without disabilities; adults with mobility limitations were more likely to have hypertension than adults without disabilities (adjusted prevalence ratio: 1.23; 95% confidence interval: 1.16–1.32). Our results suggest that adults living with disabilities are an important subpopulation to include in hypertension reporting and intervention efforts.

## Objective

Hypertension is a key treatable risk factor for cardiovascular disease, a leading cause of illness and death (1). It affects approximately 30% of US adults (2). An Institute of Medicine (IOM) report states that increasing analysis and reporting on understudied subpopulations can improve awareness, treatment, and control of hypertension (3).

Certain subpopulations, such as racial and ethnic minorities, people of low socioeconomic status, and older people are at high risk for hypertension (3,4). More than 56 million people in the United States have a disability (5) and experience health disparities (6), yet hypertension among this group has not been well researched. Our objectives were to estimate hypertension prevalence by disability status and type and assess the association between disability and hypertension using nationally representative data.

## Methods

We used data from the 2001–2010 National Health and Nutrition Examination Survey (NHANES). NHANES is a multistage probability sample of the US civilian noninstitutionalized population that combines interview data with clinical information obtained from physical examinations to assess the population’s health and nutritional status (7). We limited our analyses to participants aged 20 years or older and excluded pregnant women (n = 1,011) and participants who had missing information on either disability (n = 221) or all 3 blood pressure measurements or hypertension medication use (n = 2,690), yielding a final sample size of 23,800.

We defined hypertension as an average systolic blood pressure of 140 mm Hg or higher or an average diastolic blood pressure of 90 mm Hg or higher, based on up to 3 blood pressure measurements, or participant report of taking blood pressure–lowering medication. We defined disability based on basic actions difficulty (8) as a limitation in 1 or more of the following domains: cognitive (difficulty remembering or experiencing periods of confusion), hearing (moderate or a lot of trouble hearing, or deaf), vision (trouble seeing even when wearing glasses or contact lenses), or mobility (some or much difficulty or unable to walk for one-quarter of a mile; walk up 10 steps; stoop, crouch, or kneel; lift or carry something as heavy as 10 pounds; walk between rooms on the same level; stand up from an armless chair; stand for about 2 hours; sit for about 2 hours; reach up over head; or grasp or handle small objects).

We used SAS-callable SUDAAN, version 11.0 (9) to account for the complex survey design. Data were weighted using examination sample weights. We calculated age-adjusted prevalence estimates of hypertension by disability status and type. We used logistic regression to obtain prevalence ratios for the association between disability status and type and hypertension adjusted for age, sex, race/ethnicity, education level, income-to-poverty ratio (calculated by dividing family income by the federal poverty level specific to family size, year, and state), health insurance status, and times received care in the previous 12 months.

## Results

Prevalence of hypertension among US adults aged 20 or older was 30.0% (95% confidence interval [CI], 29.2%–30.8%). The prevalence of disability overall was 37.9% (95% CI, 36.9%–38.9%). Prevalence of disability types ranged from 6.3% (95% CI, 5.9%–6.8%) for cognitive limitation to 25.6% (95% CI, 24.7%–26.5%) for mobility limitation. Respondents were primarily aged 20 to 44 years of age (48.0%), were non-Hispanic white (71.2%), had at least a high school education (81.1%), and had some type of health insurance (80.9%) (Table 1).

**Table 1:** Study of Relationship Between Hypertension and Disability Type: Characteristics of Participants (N = 23,800), National Health and Nutrition Examination Survey, 2001–2010^a^

Characteristic	Unweighted n^b^	Weighted % (95% CI)
**Hypertension**	8,900	30.0 (29.2–30.8)
**Disability**		
Any disability^c^	10,766	37.9 (36.9–38.9)
Cognitive limitation	2,008	6.3 (5.9–6.8)
Hearing limitation	1,937	6.6 (6.3–7.0)
Vision limitation	4,918	17.7 (17.0–18.5)
Mobility limitation	7,851	25.6 (24.7–26.5)
**Age, y^d^**		
20–44	9,895	48.0 (46.6–49.4)
45–64	7,727	35.1 (34.0–36.2)
≥65	6,178	16.9 (16.0–17.8)
**Sex**		
Male	11,981	49.2 (48.6–49.7)
Female	11,819	50.9 (50.3–51.4)
**Race/ethnicity^e^**		
Non-Hispanic white	12,009	71.2 (68.4–73.8)
Non-Hispanic black	4,720	10.9 (9.6–12.5)
Mexican American	4,494	8.0 (6.7–9.5)
**Education**		
Less than high school	6,945	18.9 (17.7–20.0)
High school graduate/GED	5,749	25.2 (24.3–26.2)
Some college/associate’s degree	6,463	30.4 (29.5–31.4)
College graduate or higher	4,611	25.5 (23.9–27.1)
**Income-to-poverty ratio^f^**		
<1.00	4,176	13.1 (12.3–14.1)
1.00–1.99	5,851	20.6 (19.6–21.6)
2.00–4.99	8,072	41.6 (40.4–42.9)
≥5.00	3,960	24.7 (23.2–26.2)
**Health insurance**		
Medicare	6,265	18.6 (18.2–19.0)
Private	10,049	55.0 (53.7–56.3)
Public	2,033	7.3 (6.8–7.8)
Uninsured	5,272	19.2 (18.2–20.2)
**Times received care in past 12 months**		
0 or 1	8,218	36.0 (35.1–36.8)
2 or 3	6,186	27.1 (26.4–27.9)
4 or more	9,381	37.0 (36.1–37.8)

Abbreviation: CI, confidence interval; GED, general educational development.

a Age-adjusted to the 2000 US standard population by direct method using age groups 20–44, 45–64, and ≥65.

b Some categories do not sum to 23,800 due to missing responses.

c Disability types are not mutually exclusive and unweighted sample sizes will not sum to the total for “Any disability.”

d Not age-adjusted.

e Due to changes in oversampling rates across survey cycles, estimates for Hispanics could not be produced; race/ethnicity was categorized as non-Hispanic white, non-Hispanic black, and Mexican American. Data on respondents reporting “other” race/ethnicity are not presented but are included in total estimates.

f Calculated by dividing family income by the federal poverty level specific to family size, year, and state.

Age-adjusted prevalence of hypertension was significantly higher among respondents with disabilities than among those without (34.2% vs 26.9%; *P* < .001). By disability type, the prevalence of hypertension ranged from 29.7% (95% CI, 27.0–32.5) among adults with hearing limitation to 39.1% (95% CI, 37.4%–40.8%) among adults with mobility limitation (Table 2). With the exception of adults with hearing limitation, the prevalence of hypertension was significantly higher among adults with each disability type than among adults without any disability.

**Table 2:** Age-Adjusted^a^ Prevalence of Hypertension and Adjusted Prevalence Ratios^b^ of Hypertension, by Disability Status and Type, Adults Aged 20 Years or Older, National Health and Nutrition Examination Survey, 2001–2010

Disability Status	% Hypertension (95% CI)	Hypertension APR (95% CI)
Any disability^c^	34.2 (33.1–35.4)^d^	1.13 (1.08–1.20)
Cognitive limitation	36.4 (33.8–39.1)^d^	1.16 (1.05–1.28)
Hearing limitation	29.7 (27.0–32.5)	0.99 (0.91–1.09)
Vision limitation	32.9 (31.5–34.4)^d^	1.12 (1.04–1.20)
Mobility limitation	39.1 (37.4–40.8)^d^	1.23 (1.16–1.32)
No disability	26.9 (25.8–28.1)	1 [Reference]

Abbreviation: CI, confidence interval; APR, adjusted prevalence ratio.

a Age-adjusted to the 2000 US standard population by direct method using age groups 20–44, 45–64, and ≥65.

b Adjusted for age, sex, race/ethnicity, education level, income-to-poverty ratio, health insurance status, and times received care in the previous 12 months.

c Disability types are not mutually exclusive.

d *P* < .001 when compared with “No disability”; *P* values determined using a 2-tailed *t* test.

After controlling for sociodemographic variables, adults with disabilities had a 13% higher prevalence of hypertension than adults without any disability (adjusted prevalence ratio [APR] = 1.13; 95% CI, 1.08–1.20). There were significantly higher prevalence ratios for adults with most types of disability than for adults without any disability, the highest being for adults with mobility limitation (APR = 1.23; 95% CI, 1.16–1.32) and the lowest for adults with vision limitation (APR = 1.12; 95% CI, 1.04–1.20). There was no significant difference after adjusting for sociodemographic variables in the prevalence of hypertension between adults with hearing limitation and adults without disability (Table 2).

## Discussion

We found that adults with disabilities are more likely to have hypertension, even after controlling for sociodemographic and health care access indicators. This disparity, and the variation by disability type, may in part be explained by behavioral risk factors such as obesity, smoking, and physical inactivity that co-occur with or worsen the effects of hypertension (1,10) and that disproportionately affect people with disabilities (6,11). For example, the prevalence of movement difficulty is 1.5 to 2 times higher among obese adults than among adults with a normal body mass index (12).

There are several limitations to our analysis. First, NHANES data are limited to the community-dwelling population and exclude people who reside in institutional settings. This exclusion may result in an underestimate of hypertension prevalence among adults with a disability. Second, the data collection methods are cross-sectional and do not allow us to draw inferences about the causality between disability and hypertension. Third, blood pressure was measured during a single visit, which may overestimate or underestimate hypertension prevalence (1). However, this factor would likely affect people with and without disabilities in a similar manner and should not have affected the magnitude of the disparities noted here.

Citing the public health significance of hypertension, a recent IOM report noted the importance of identifying at-risk subpopulations (3). To our knowledge this is the first study to describe hypertension by disability status and type in a nationally representative US sample. Consistent with recent studies among the general population, we controlled for sociodemographic and health care access indicators (2). Future studies should explore behavioral factors that could also be related and differences in treatment and control of hypertension for adults with disabilities. Our results suggest that adults living with disabilities are an important subpopulation to include in reporting and intervention efforts that work toward reducing hypertension prevalence.
